# Pragmatics in language change and lexical creativity

**DOI:** 10.1186/s40064-016-1836-y

**Published:** 2016-03-17

**Authors:** Keith Allan

**Affiliations:** Monash University, Melbourne, Australia; University of Queensland, Brisbane, Australia

**Keywords:** Euphemism, Dysphemism, Orthophemism, Metaphor, Metonymy, Figurative language

## Abstract

This essay examines language change and linguistic creativity as revealed by remodelling, especially as a source for euphemisms and euphemistic dysphemisms and as a function of verbal play. Within the scope of this essay, there are predominantly two ways in which novel terms and expressions are created leading to language change: formally through remodelling and semantically through figurative language. Consider some of the words for nakedness. There is the orthophemistic term *nude*, from Latin *nudus*, often used of photographic or painted representations of naked women and, much more rarely, of a naked man—hence the marked term *male nude*. Whether *a nude* is artistic or pornographic depends on the viewer belief. A colloquial Australian euphemism for being *in the nude* is *in the nuddie*. Other euphemisms include *as nature intended, in one’s birthday suit, in the altogether*, and *in the buff* (⇐*buff*[*alo*] *leather, buff skin* transferred to humans). Being naked is captured by the dysphemism *bare*-*arsed* and the more euphemistic *butt / buck naked* in which *buck* ⇐ *butt*. The orthophemistic term *stark naked* and the connected colloquial euphemism *starkers* also arose by replacing a final /t/ with a /k/: *stark* ⇐ *start* “tail, arse”. *Nudists* like to go about in the open air without clothes on and, being *as nature intended* when in natural surroundings, are euphemistically called *naturists*. Such expressions display folk-culture in a remarkable inventiveness of metaphor and figurative language sourced in the perceived characteristics of whatever is being talked about. For instance, terms for tabooed objects and events provide ready-made material for the dysphemistic language of curses, insults, epithets, and expletives. The essay shows that X-phemisms (orthophemisms and/or euphemisms and/or dysphemisms) are motivated by a speaker/writer’s want to be seen to take a certain stance by upgrading, downgrading, obfuscating, and deceiving; and they extensively manifest indulgence in verbal play. Although the discussion focuses on English, the categories illustrated occur across the world’s languages, and many of them are significant for the study of language change.

## Background

The (pragmatic) application of context and world knowledge to the creation and interpretation of novel expressions is blindingly obvious when we survey a host of examples of expressions developed in the interests of verbal play or taboo avoidance. These examples of linguistic creativity give rise to language change in the area of lexis. Many people double take when first confronted by the designer label *FCUK* because one cannot avoid associating *FCUK*—based on the initial letters of the phrase ‘French Connection United Kingdom’– with the highly salient obscene term *fuck*.^1^ This re-shaping of what is seen to match something familiar and meaningful is irresistible. Thus no fluent speaker of English has any trouble interpreting (1), though at first sight it may appear nonsense.

(1): Aoccdrnig to a rsecherear at an Elingsh uinervtisy, it deosn’t mttaer in waht oredr the ltteers in a wrod are, the olny iprmoetnt tihng is that frist and lsat ltteer is at the rghit pclae. The rset can be a toatl mses and you can sitll raed it wouthit porbelm. Tihs is bcuseae we do not raed ervey lteter by itslef but the wrod as a wlohe

When a speaker/writer remodels a language expression with the intention of communicating, a fluent listener/reader does not normally have too much trouble recognizing the intended meaning. Taking context into account and working on a system of analysis-by-synthesis we match the misspelled words in (1) with their normal forms.

More challenging are literary artifices like John Lennon’s *A Spaniard in the Works*; here’s a sample.

(2): ‘I really don’t know woot to mak of these,’ said Norman, as he sorted through him Chrimbas posed. ‘It seem woot I git mower litters und parskels than woot I know peoples, it suplizeses moi moor et moor each yar, as moor on these pareskle keep cooming. I really doon’t knaw whew all they body are—seddling ik all this.’ (Lennon 1965: 66)^2^

Somewhat more challenging than (2) because it lacks phonetic similarity as a guide is Lewis Carroll’s *Jabberwocky* (Carroll 1872) and far more challenging is *Finnegans Wake* (Joyce 1939) with its erudite morphological innovations.

Making sense of texts like (1) and (2), from both aspects of creation and interpretation, is similar to what we find with expressions like *Golly!* or *Gosh!* where a profane use of the expletive *God!* would make perfectly good sense; they can communicate effectively. And the same is true for *Cor! Cor lumme! Gorblimey! Gordonbennet! Gordon’ighlanders! Goodness (knows)! (Good) gracious! For goodness’ sake!* Such remodellings of the word *god* are deliberate ploys to avoid explicit profanity (i.e. careless irreverence for the deity or other religious icons). This avoidance displays a certain stance: an altruistic desire not to offend and/or the face-saving aspiration not to seem to be offensive.

## Euphemistic dysphemisms

The quaint archaisms *’Od’s life! ’Sblood! ’Sbodlikins! Zounds! Gad! Gog! Cock! Cod!* were generated for the same reason, though at a time when a speaker/writer might also be heavily fined for taking the Lord’s name in vain. Other euphemistic dysphemisms of this nature are *(Oh) Lord!, Lordy! Lawdy! La! Land’s sake!* and *Heavens (above)!* or *Heavens to Betsy!* These were commonly used by Victorian ladies (Montagu 1968: 225) and many would have been aware that though such expressions avoid explicit profanity, they function as oaths; but perhaps some, like Ophelia in Shakespeare’s *Hamlet*, were naïvely unaware of doing so.

Ophelia, whether dissembling or in innocent ignorance, utters the mild oaths: ‘la’ ⇐ *Lord*, ‘by Gis’ ⇐ *by Jesus*, ‘by Saint Charity’ (that there never was such a saint did not stop people swearing by her), ‘fie’ ⇐ [*by my*] *faith*, ‘by Cock’ ⇐ *by God*. Typically, Shakespeare weaves in several sexual innuendos: the juxtaposition of ‘young men’, ‘come’, ‘Cock’, ‘before you tumbled (the ostensible meaning is “tousled”) me, you promised me to wed’ yields the meaning confirmed by the man’s answer, “before you bedded me you promised to marry me, and now you won’t”; it is a reference to the ageless double-standard—the girl who agrees to sex before marriage is not chaste enough to wed. To fully appreciate the rich nuances of meaning in (3) the reader/listener, like Shakespeare himself, needs to draw on sources beyond the semantics or literal meaning of the text and augment it having regard to its context and to world knowledge, which Shakespeare is taking to be common ground (Allan 2013; Clark 1996; Grice 1981; Lee 2001; Lewis 1969; Stalnaker 1973, 2002). The same applies to a lesser degree to the creators and interpreters of the remodelled profanities illustrated earlier.

Chaucer wrote:

(4): ‘Ye,’ quod the preest, ‘ye, sire, and wol ye so?
  Marie! there-of I pray yow hertely.’ (Chaucer 1396 Canon’s Yeoman’s Tale 1061f)

Mary, mother of God, is the source for the Canon’s Yeoman’s ‘Marie’ later remodelled to *Marry* as in *Marry forbid!* and *Marry come up!.* Rather similar in meaning to the expostulary *Marry!* were *Fie!* and *Fackins!* remodelled from *Faith!* all of them having much the same force as today’s profane *God!.* These are more archaic than *Holy Mary!* and *Holy mother!* whence, probably, *Holy cow!* and the double dysphemisms *Holy shit! Holy fuck!*. Less profane than *Holy Mary!* are *Holy Moses!Holy mackerel!*—these are real euphemisms along with such as *Holy toot!Holy demolition, holy Halloween, holy hallucination, holy hairdo, holy sardine*—most of which are obviously jokey (see https://en.wikipedia.org/wiki/List_of_exclamations_by_Robin). *What in Hades?!* is perhaps polite variation on *What in hell?!* Curiously, although *What the deuce?!* is analogous to *What the dickens?!* and *What the devil?!*, ‘deuce’ here derives from the Norman French oath *Deus!* “God”. *What the dickens?!* avoids calling up by name the malevolent spirit of *Old Nick,Old Harry, Old Bendy, Old Bogey, Old Poker, Old Roger, Old Split*-*Foot, the Old Gentleman, Old Billy*. Confounding someone or something was euphemized in *Od rabbit it* from *God rot it!*—which lives on in *Drat it!* or simply *Drat!.* There was always the explicit *Damnation!* remodelled to *Tarnation!* as *Damn!* is remodelled to *Darn!* and *Dang!* All of these can be classed euphemistic dysphemisms: they are phonetically similar to the dysphemism they replace and yet have a similar communicative function to that dysphemism. As one participant in a survey admitted:

(5): If I saw a euphemism written down I guess it would be the same as seeing a swearword as far as I’m concerned, because I know what it would represent. (Millwood-Hargrave 2000: 43)

The term *profanity* once applied only to (supposedly) inappropriate use of religious terms; it has expanded its domain to another kind of ‘bad language’ namely, obscenity. *Shit!* gets remodelled to the euphemistic dysphemisms *Sugar!Shoot!Shucks!* and *Shizzle!*. A *fuck up* becomes a *foul up*; the adjective and gerund *fucking* becomes *mucking, freaking, frigging*. The insulting appellation *bastard* is remodelled to *basket* and *bugger* ⇒ *begger*. Historically *tydbit* was used earlier than *titbit*. Today the use of *tidbits* for *titbits* (primarily in America) seems unnecessary because Britain has a variety of birds named *tits* (Paridae) which creates no politeness or appropriacy problems—though it does allow for sad jokes like ‘Twenty WRNS walked into the cold store and forty blue tits came out’ (WRNS, homophonous with *wrens*, is the acronym for Women’s Royal Naval Service). *Cunt* is reformed into *cooch, coochie, hoochie*-*coochie* and *oochamagoochi*. Anglo-Americanized pronunciation of the Hispanic California place name *Tres Pinos* (‘three pines’) would normally be /ˈtrɛs ˈpinoʊs/, making *Pinos* almost homophonous with *penis* /ˈpinəs/ and fully homophonous if the final unstressed syllable of *Pinos* is reduced to /–nəs/. To avoid embarrassment, *Pinos* is phonologically dissimilated to the un-Spanish-like /ˈpainoʊs/. Before *coney* “rabbit” dropped out of use in the late nineteenth century, it was often pronounced /ˈkʌnɪ/ (rhyming with *honey*). However, this became the standard pronunciation for the homonymous word meaning “cunt” (which lives on in *cunnilingus*) and so the “rabbit” sense was remodelled to /ˈkoʊnɪ/, as it remains in *Coney Island*. There is similar dissimilation with the word *twat* which (in Britain at least) is usually pronounced /twɒt/ to mean “idiot” and /twæt/ to mean “vagina”. Pragmatic awareness of what may be interpreted as impolite or inappropriate leads to modification of what is said, leading to the coining of new forms.

## Contractions

Another path to remodelling is contraction of one kind or another. When *underpants* are referred as *pants* it is a fore-clipping, and when as *undies* it is an end-clipping.^3^ Clippings imply what Recanati (2002) calls ‘unarticulated constituents’.*’Od’s life* and *’sblood* are fore-clipped as is the archaic *nation* ⇐ *damnation* (Grose et al. 1811). *Gee* is end-clipped *God* or *Jesus* and *jeeze* from the latter; *bra* is end-clipped *brassiere* and *pee* ⇐ *piss*. Clipping is a form of contraction often referred to as ‘abbreviation’. Both acronyms and alphabetisms are constructed from the initial letters of phrases but acronyms differ from alphabetisms by being pronounced like words rather than a list of letters. Thus *snafu* /ˈsnɑfu/ is an acronym for “Situation Normal, All Fucked (euphemistically, ‘Fouled’) Up”. On the other hand *S.O.B.* /ˈɛs oʊ bi/ is an alphabetism for “Son-Of-A-Bitch”; *RTFM* is an alphabetism for “Read The Fucking Manual”. The spoken forms of both *Gee* and *pee* could be classed as alphabetisms of single words because of their homophony with letters *G* and *P*, but they are also end-clippings, and their written forms make them look more like acronyms! So *Gee* and *pee* fall into three categories, of which the least controversial is end-clipping. (The fact that *Gee* and *pee* fall into three categories is not a source for analytical despair: the three categories support one another to deliver meaning).

When *f*—is printed in place of *fuck* we have a case of end-clipping; if we see f*** it is a case not only of end-clipping but also quasi-omission in that each missing letter is replaced one-for-one by a non-alphabetic symbol. We also find quasi-omission in the middle of words and the omitted letters may or may not be indicated severally, e.g. *d*—*n* (⇐*damn*), *d*—*nation*; *w*—*e* or *wh*—*re* (⇐*whore*) (Fielding 1749); and *f*—*k* and *s*—*t* (*The Age*, Melbourne, October 17, 2002).

(7): Cauliflower. […] Also the private parts of a woman; the reason for which appellation is given in the following story: A woman, who was giving evidence in a cause wherein it was necessary to express those parts, made use of the term cauliflower; for which the judge on the bench, a peevish old fellow, reproved her, saying she might as well call it artichoke. Not so my lord, replied she; for an artichoke has a bottom, but a **** and a cauliflower have none. (Grose et al. 1811)

This, together with the appearance of **** in other entries of Grose’s *Dictionary of the Vulgar Tongue*, leaves no doubt that the four asterisks are a synonym for that four-letter word that he otherwise calls ‘the monosyllable’ and in today’s media is called ‘the c-word’.^4^ It is highly significant that *the monosyllable, the c*-*word*, and *the f*-*word* all take the definite article, *the*: they are as much proper names as *the Himalayas* and *the Pope*, immediately recognizable to the normal speaker of English despite the fact that, though there is only one set of Himalayas and (today) only one Pope, there are thousands of monosyllables and thousands of words beginning with *c*- and *f*-. It is not merely context that leads underspecified expressions like *the c*-*word* to be immediately understandable; it is the shared common ground in the salience of the lexicon associated with sex, micturition, and defecation. Deformations like c*** and *f*–*k* draw attention to the word’s being unprintable and thereby saliently mark the obscenity.

The spoken counterparts to dashes and asterisks are non-lexical substitutions like *mhm, er*-*mm*. For example, in a novel published in 1926, a lady says on discovering some French novels on her friend’s table:

(8): ‘This is a little—h’m—isn’t it?’
  ‘I read those things for their exquisitely polished style; the subjects escape me.’
  (Pinero 1900: 116f)

The answer is classic, and as unbelievable as the heterosexual man who says he buys *Playboy* for the articles and never ogles the photos of attractive nude nubile women.

Full-omissions are also a kind of clipping. There is *Gracious!* either fore-clipped from *Good gracious!* or end-clipped *Gracious God!*; all three make profane reference to God’s graciousness, but *Gracious!* makes full omission of the name of God. Among full omissions, end-clippings seem to be most common. E.g. *the ladies / gents* omits *lavatory*; and *I need to go* can also be understood to omit *to the lavatory*. The commonly used jibe *There’s the pot calling the kettle black* omits *arse* from the end (anyone who has cooked over a wood or coal fire knows that pots get black bottoms). A magazine advert for Vagisil™ Feminine Itching Medication modestly omits to mention the location of the feminine itching—perhaps relying on the product name to aid the unimaginative.

(9): This instantly-soothing medication relieves external feminine itching as easily as aspirin relieves a headache.
  That’s good news because minor feminine itching is about as common as a headache—caused by everyday things like jogging, pantyhose, even normal perspiration.

All these examples of full-omissions fall within the category of general-for-specific euphemisms. All such X-phemisms rely on pragmatic augmentation of what is said to elucidate what is meant. Such pragmatic expansion is anticipated by whoever coined the expression initially and by all who subsequently utter it and also, of course, by those who correctly interpret what is said.

## Metonymy and synecdoche

General-for-specific (whole-for-part) euphemisms, together with part-for-whole X-phemisms are sometimes discussed in terms of ‘metonymy’ and ‘synecdoche’. The meanings of the terms *metonymy* and *synecdoche* overlap to the extent that there is little point distinguishing between them. The confusion arises because they are almost synonymous: etymologically *metonymy* means “one name exchanged for another” and *synecdoche* “to take with something else”. The advert for Vagisil™ Feminine Itching Medication, quoted in (9) uses ‘feminine itching’ as a general-for-specific euphemism; that advert was coupled with another in which ‘feminine moisture’ is another general-for-specific euphemism:

(10): **Feminine moisture** end it now and stay fresher all day
  Now stay drier, feel fresher all day with VAGISIL™, the first Feminine Powder with a totally unique formula to solve wetness problems.

Another such euphemism is the use of *person* for ‘penis’ in legal discourse (this example is also a one-to-one substitution).^5^ The use of *nether regions* and *down there* for “genitals” invokes the-general-area-for-a-specific-area-within-it. *Go to bed* as a euphemism for “copulation” invokes the-usual-location-where-a-specific-event-takes-place. It is clear that the second clause of *Harry and Sally went to bed, but not together* cancels some relevant implicature that is normally expected to augment what is actually said in the first clause. *Go to bed (together)* is a member of a large set of similar *go to* expressions which underspecify the meaning, yet succeed in referring appropriately by invoking a semantic frame or script,^6^ e.g. *go to a restaurant* (being seated, selecting food, being served food, paying for the meal, etc.) and on more delicate topics *go to the doctor / hospital, go to the bathroom /toilet / loo, go to the block / gallows / electric chair* all omit reasons for going. The-maximally-general-and-non-specific-for-something-specific strategy for euphemism is exemplified in former U.S. President Richard Nixon’s references to *prething* and *postthing* (where ‘thing’ = “the 1972 Watergate break-in” which brought him down), the use of *thingummybob* for “penis” (or whatever), and expressions like *the you*-*know*-*what* to denote almost anything that can be readily inferred from context. Rather similar was (is?) the use of *inexpressibles* or *unmentionables* and perhaps *smalls* for underclothing; also Grose’s use of *the monosyllable* meaning “cunt”.

Many general-for-specific euphemisms are understatements, e.g. *thing* for whatever (“Watergate break-in”, “genitals”, “cancer”) or *deed* for “act of murder” (or whatever); but also *anatomically correct dolls* for “dolls with sexual organs”; and expressions like *companion*, *friend*, *this guy I’m seeing* and even *lover* for “regular sexual partner”. At one time the French verb *baiser* meant “kiss” (*embrasser* has replaced it) and has come to mean “screw, fuck”—the transition shows a nice euphemistic understatement which in this case is also a part-for-whole euphemism.

Part-for-whole euphemisms include *powder my nose* and *spend a penny*^7^ for “go to the lavatory”; and *I’ve got a cough* may occasionally ignore the stuffed up nose, post-nasal drip, and running eyes. Afrikaans *ghat*, originally “hole”, is used in much the same way as British or Australian *bum*, American *fanny*. The now archaic (?) reference to a woman as *tit* was usually dysphemistic but could be affectionate; it may be a part-for-whole dysphemism, or a transfer from the tit as *bird* (genus *Parus*) metaphor. Referring to a girl-friend as a (*bit of*) *cunt / ass* is dysphemistic; as is calling someone an *arsehole* / *asshole*. So, the use of offensive body-part terms as insults often fits among this set of X-phemisms. Most part-for-whole metonymies are orthophemistic: *The pen is mightier than the sword; We are doing Shakespeare next week; The Caesar salad wants a glass of beer.* All these X-phemisms require pragmatic input to augment the bare words uttered (the locution).

## Euphemistic jargon

There is a whole batch of euphemisms for avoiding the mildly distasteful, upgrading what is favoured and downgrading what isn’t. Some euphemisms are downright deceptive and others deliberately obfuscatory. In 1997 Australian Prime Minister John Winston Howard introduced the compound *non*-*core promise* into the English language; it is defined by www.disinfopedia.org as “a promise not kept, in most cases a lie from the start”. It is notable that many upgrading and deceptive euphemisms are circumlocutions; they are comparatively verbose and sometimes obfuscatory politically correct expressions that smack of jargon.

(11): Bribes, graft and expenses-paid vacations are never talked about [in the US House of Representatives] on Capitol Hill. Honorariums, campaign contributions and per diem travel reimbursements are. (*Time Australia* April 17, 1989: 36)

In (11), the terms *honorarium*, *campaign contributions*, and *per diem travel reimbursements* are used as alternatives to the dispreferred expressions *bribes*, *graft*, and *expenses*-*paid vacations*—because they have positive instead of negative connotations. Take the example of a landlady who prefers to say she has *paying guests* rather than *lodgers* because, to her mind, *paying guest* has fewer negative connotations than does *lodger*. So does the euphemistic understatement *sleep* for “die”. When Janet Jackson’s right breast was revealed by fellow singer Justin Timberlake at the NFL Super Bowl show on February 1, 2004, it was described as a *wardrobe malfunction*.^8^ On the financial markets, when currency is taking a tumble, dealers and economists talk of it having *a substantial downside risk potential*; falling stocks *go south*; rising stocks go *up*, but never *north* (except in jest). In 2004 an MBA Alumni event charged a so-called ‘Investment’ of $50 for alumni and $65 for guests.^9^ An author who reads in a publisher’s letter *After careful consideration we have regretfully concluded that your manuscript falls outside the scope of our current publishing program* interprets it to mean ‘We don’t want to publish your manuscript.’ *We’ll have to let you go* replaces “You’re fired”, even when *dehiring* is merited. Companies *restructure* and *downsize*, they don’t *lay off* employees, let alone *sack* them.

(12): Fifteen employees at Clifford of Vermont, Inc. weren’t laid off. ‘This was not a cut back nor a lay-off. It was a career-change opportunity,’ said John McNulty, president. *Valley News* (Conn.), 3 May 1990. (Quarterly Review of Doublespeak 17/1, 1990: 1)

McNulty was just *eliminating redundancies in the human resources area* by *releasing* these employees. Decisions are made about *targeted voluntary separation*, which seems in fact to be involuntary. People arrested but not yet charged are *helping the police with their enquiries*. A barrister’s *refresher* is “the fee for the second and each subsequent day of a hearing”. *Adult videos* are pornographic. And a *starter home* or a *cosy cottage suitable for renovation* is so much more enticing than a *small dilapidated dwelling*. *Life insurance* supposedly insures the value of your life but, ironically, is in effect “payout to your estate on your death”. If a soldier is hit by *incontinent ordinance* (a kind of *friendly fire*) s/he may suffer a *ballistically*-*induced aperture in the subcutaneous environment* or worse. If you jump out of a 10th-storey window, you’ll suffer *sudden deceleration trauma*. The final remark on the hospital chart of a case of *negative patient care outcome* was ‘Patient failed to fulfil his wellness potential’ (Lutz 1989: 66); it is not reported whether this resulted from *therapeutic misadventure*. Calling vinyl *vegetarian leather* may be kind of a joke; not so the Third Reich’s *final solution* nor *ethnic cleansing*. Hopefully, the *strange fruit* that *decorated the cottonwoods* in the American south will never be seen again (www.youtube.com/watch?v=h4ZyuULy9zs). On a lighter note, whereas simple dieting would involve you in *negative expenditure*, you might be willing to pay for *nutritional avoidance therapy*. A *sanitation engineer* sounds more exalted than a *garbage collector*; a *vermin control officer* has replaced the *ratcatcher*. The *night watchman* has become a *night entry supervisor*. A *preloved* object sounds more attractive than a *second*-*hand* or *used* one does; they can be found in an *opportunity shop*, which specializes in *reutilization marketing*. One is, at best, *comfortably off* oneself; other people are *wealthy* or even *filthy rich*. *A categorial inaccuracy* or *terminological inexactitude* is “a lie”; *the person I am wont to refer to by the perpendicular pronoun* is “I/me”, and *little girl’s room* is “(female) toilet”. Education departments refer to *those on the lower end of the ability scale*, to *low ability subjects* and *educationally disadvantaged / challenged* groups (see Peterson 1986: 54). Perhaps litotes like *he’s not unintelligent* should be included here along with ironic hedges like *He’s not very bright* meaning “he’s as thick as two short planks”. Few such circumlocutions warrant dictionary entries in their own right. Some paraphrase a short expression in a kind of semantic analysis in which the meaning of the taboo term is unpacked and its components listed: e.g. *ratcatcher* becomes *vermin control officer*; *pet* becomes *companion animal*; *rape* becomes *criminal sexual assault* or *a serious offence against a woman*; *urine* becomes *excrementitious human kidney fluid*; *faeces* becomes *solid waste matter*; and *pus* is *viscous matter of a wound*. Many language expressions castigated as jargon are paraphrases of this kind.

Hyperboles are found in euphemisms like *flight to glory* meaning “death”, or *villa in a premier location by the bay* referring to a dilapidated artisan’s cottage, five streets away from the bay, or *Personal Assistant to the Secretary (Special Activities)* for “cook”, which ‘illustrates a basic rule of bureaucracies: the longer the title, the lower the rank’ (Rawson 1981: 11)—presumably to upgrade the lower ranks in at least this one inexpensive respect. Hyperbole is a common euphemistic device in obituaries and epitaphs to assist those left alive in coping with the pain of loss and the fear of dying (see Allan and Burridge 1991; Crespo-Fernández 2006, 2011).

When Grose et al. (1811) use *the monosyllable* as a euphemism for *cunt*, the phrase simply acts as a substitute; compare the use of *bottom* for “arse/ass”, *smalls, scanties, frillies* or *jocks* for “underwear”; *casket* for “coffin”; *break a leg* instead of “Good luck” in the theatre; the somewhat archaic *a bit of the how’s your father* for “have sex, copulate”; the *adult services* advertised in the yellow pages and newspapers are “sexual services”. The fields of X-phemism, jargon and slang exhibit huge numbers of substitutions.

## Execration in a language other than English

Language usage suggests that other peoples are more depraved than one’s own nation. For instance, in 1990, a Tucson (Arizona) shop selling so-called *adult* (i.e. pornographic) *books and videos* described itself as a *Continental Adult Shop*, the European continent being, for the English speaking world of the nineteenth and twentieth centuries, the apocryphal home of depravity. The English idiom *excuse my French* derives from the kind of xenophobic dysphemism found in the languages of all human groups.

(13): ‘Why, supposing that universities are organized like businesses, with a clear division between management and labour, whereas in fact they’re collegiate institutions. That’s why the whole business of the cuts has been such a balls-up. Excuse my French, Robyn.’
  Robyn waved the apology aside. (Lodge 1989: 344)

Among many other examples, English includes *Dutch courage*, calling someone a *Jew* “stingy”, a *Nazi* “a person who displays amoral, dictatorial characteristics”, and once there was the *Spanish pox*. But it is the French, long-time enemies of the English, who cop most of the sex-related dysphemism: *French pox* was an alternative to *Spanish pox*, *French kiss*, *French letter*^10^ (or as the French call it the *capote anglaise* “English hood”, *redingote anglaise* “English overcoat”^11^), *give French* “perform fellatio”, *French novels* (supposedly pornographic). Borrowings from the French language include the seventeenth–eighteenth century use of French *mot* “word” as a euphemism for *cunt*; giving rise to *mot* (also spelled *motte*) “piece of arse, bit of cunt, dysphemistic reference to a woman”, and the more recent *masseuse* for “prostitute”, *lingerie* for “women’s underclothing”, *brassiere* (euphemized, perhaps, in the abbreviation *bra*), *po* for “chamber pot” from French *pot* /po/, *toilet* a euphemism from French *toile* “cloth”, *gauche* “clumsy” (from the nearly universal denigration of left-handedness), *matériel* for “armament and ammunition”, *sortie* for “a sallying forth by a military unit”. The motivation for borrowing all of these terms was euphemism and, for most, there is a native English alternative. An exception is *brassiere* where alternatives are usually dysphemistic: *tit*-*covers, breastplates, over*-*shoulder boulder*-*holders* (compare the Viennese *Mirabellenetui* “plums-case”).

In his Dairy, kept from January 1, 1660 until May 31, 1669, Samuel Pepys slipped into a mixture of Latin, French, Spanish, Italian and pseudo-romance when reliving his sexual exploits (Pepys 1976). ‘The garbled foreign phrases he often used for sexual incidents had something to do with concealment perhaps, much more with his pleasure in marking off sexual experiences with special words and so heightening the excitement of reliving them’ (Tomalin 2003: 266). Some examples:

(14): *nulla puella negat* “the girl refused nothing”^12^;
  my mind un peu troublé pour ce que j’ai fait [“my mind a bit troubled by what I did”] today. But I hope it will be la dernière de toute ma vie [“the last of my whole life”].^13^
  poner my digito [“put my finger”] in her thing, which did her much pleasure; but I pray God that ella [“she”] doth not think that yo [“I”] did know before—or get the trick of liking it.^14^
  walked (fine weather) to Deptford and there did business and so back again; walked, and pleased with a jolie femme that I saw going and coming in the way, which yo could aver sido contented para aver [“would have been very contented to have”] stayed with if yo could have ganar acquaintance con ella; but at such times as those I am at a great loss, having not confidence, ni alguno [“not any”] ready wit.^15^
  Je besa her venter [“kissed her belly”] and cons [“vulva”] and saw the poyle [“(pubic) hair”] thereof.^16^
  did tocar mi cosa con su mano [“take my penis in her hand”] through my chemise, but yet so as to hazer me hazer la grande cosa [“but nonetheless caused me to have an orgasm (literally, great thing)”].^17^
  grand envie envers elle, avec vrai amour et passion [“strong feelings for her, with true love and passion”] … [but she] would not laisser me faire l’autre [“let me do the other”] thing, though I did what I pouvais [“could”] to have got her à me laisser [“to let me”].^18^
  I did read through *L’escholle de Filles* [“The Girls School”]; a lewd book, but what doth me no wrong to read for information sake (but it did hazer my prick para stand all the while, and una vez to decharger [“come”]); and after I had done it, I burned it, that it might not be among my books to my shame.^19^
  did begin to tocar [“touch”] the breasts of my maid Jane, which ella did give way to more than usual heretofore, so as I have a desire to try more than I can bring it to.^20^
  I did give her good advice and beso la, ella [“kissed her, she”] weeping still; and yo did take her, the first time in my life, sobra mi genu and poner mi mano sub her jupes and toca su [“on my knee and put my hand under her skirt and touched her”] thigh, which did hazer me great pleasure; and so did no more, but besando-la [“kissing her”], went to my bed.^21^ …
  [F]irst with my hand tocar la cosa de [“touched the vagina of”] our Deb in the coach—ella being troubled at it—but yet did give way to it.^22^ … [Wife Elizabeth found him with Deb] with my main [“hand”] in her cunny.^23^

Pepys’s wife Elizabeth suffered from Bartholin’s abscess or cyst on her vulva (Tomalin 2003: 399 n.15); though treatable today with antibiotics, it was untreatable in the seventeenth century and recurred throughout her lifetime. He used a variety of euphemisms.

(15): my wife not very well of her old pain in the lip of her *chose*, which she had when we were first married.^24^
  we fear that it is my matter that I give her that causes it, it never coming but after my having been with her.’^25^

The Diary opens with a reference to the couple’s distress at Elizabeth not being pregnant:

(16): My wife, after the absence of her terms for 7 weeks, gave me hopes of her being with child, but on the last day of the year she hath them again.^26^

Elizabeth’s often difficult menstrual cycles (‘she was in great pain of those’^27^) are ‘a sad repeated message tolling through the years of the Diary under many different names, her menses, her months, her being unwell, *ses mois, ceux*-*la, moys, mois*—no doubt her own usage was French [the first language of Elizabeth’s family]’ (Tomalin 2003: 89).

C.S. Lewis, author and Christian apologist, said that to speak of the organs of sex, micturition and defecation and associated matters ‘you have to resort to the language of the nursery, the gutter, or the medical textbook’ (Tynan 1975: 185). Bataille (1992: 138) says something very similar: ‘The sexual organs and the sexual act in particular are referred to by degrading names from the jargon of the dregs of society. Those organs and acts have other names, but some are scientific and others, more rarely used and shorter lived, belong to childhood or the shyness of lovers.’ By the Middle Class Politeness Criterion (Allan 2015a; Allan and Burridge 1991, 2006), ‘the language of the gutter’ is dysphemistic. Between different generations ‘the language of the nursery’ is euphemistic (e.g. a Mandarin speaking mother may refer to a child’s penis as *xiaoji* or *jiji* “little chicken”, an Anglo-Jewish mother will refer to a child’s backside as his or her *tushy*). The ‘language of the medical textbook’ is deliberately non-titillating; it is primarily based on Latin (*expectorate, menstruate, penis, urinate*) and less frequently Ancient Greek (*diarrhoea,dysmenorrhoea*). The history of such words may be chequered: Cicero remarked that Latin *penis* “tail” was in his day an obscenity though it was once a euphemism for *mentula* (literally “sprig of mint”)—itself presumably a euphemism at an even earlier time.

The use of Latin-based synonyms provides Standard English with orthophemisms for bodily effluvia, sex, and the associated acts and bodily organs: examples are the use of *perspire* instead of *sweat*, *expectorate* instead of *spit*, *defecate* and *faeces* instead of *shit*, *copulate* instead of *fuck*, *anus* instead of *arsehole, genitals* or *genitalia* instead of *sex organs*, *vagina* instead of *cunt*, *labia* instead of *lips* (of the vulva), and so forth. Until the late twentieth century, translations of taboo terms from exotic languages, and descriptions of taboo acts, caused an author to suddenly switch from English to Latin. For instance, Hollis in *The Masai: Their Language and Folklore* translates the story of the demon Konyek: at one point in the story Konyek sits beneath a tree in which a frightened woman is hiding, causing her to tremble so much that in the Maasai story she *neisirisir ngulak*, which Hollis (1905: 137) translates as ‘Incipit mingere guttatim.’ It would be more aptly rendered for today’s English readership as “it made her piss (or, euphemistically, wet) herself”. His translation of the very brief Maasai tale*’L omon le*-*’ngai o en*-*gop* is as follows:

(17): The Story Of The Sky And The Earth
  Haec verba dicere volunt. Ut maritus supra feminam in coitione iacet, sic coelum supra terram. Ubi lucet sol et cadit imber, terra calorem recipit et humorem: non aliter femina hominis semine fruitur. [“They say that just as a husband lies on top of his wife in coition, so does the sky above the earth. When the sun shines and the rain falls, the earth receives heat and moisture: in the same way a woman is fertilized by a man.”]
  (Hollis 1905: 279)

In Lewis and Short’s *A Latin Dictionary* (Lewis and Short 1975) the meaning for *cunire* is given in Latin as ‘est stercus facere’ “have a shit” instead of English *defecate*. On such occasions Latin was euphemistically used because of the author’s prudery in not wishing to use everyday English terms, but with the added rationalization that the Latin text would be uninterpretable to the uneducated—and therefore to the young and innocent, and to many women (who were not supposed to hear, and certainly not to speak of, such things). The antithetical strategy is to use colloquial rather than more formal terms, e.g. *period* for *menstrual cycle*.

Using words borrowed from other languages to function as euphemisms is characteristic of many languages. Foreign words are salient in any text and they often signal that something out of the ordinary is going on which the reader/listener needs to figure out. On many occasions the use of a foreign expression is simply a matter of style, as with most uses of e.g. *ibidem, quid pro quo, mutatis mutandis, ad nauseam,* etc.

## Picturesque X-phemisms

(18): [Y]our daughter and the Moor are now making the beast with two backs
  (Shakespeare *Othello* I.i.114)

(18) describes what is also referred to as *tupping, covering, mounting, riding, coupling, humping, folk*-*dancing, doggy*-*dancing, horizontal dancing, horizontal jogging, jigjogging, uptails all, belly slapping, roll (in the hay)* as well as many terms of attack and terms for penetration.

The anus (*anus* is Latin for “ring”) is referred to as *ring*, *hole, brown*-*eye, date, arsehole, shithole, blurter.* A *rim job* or *rimming* is anilingus. To tell someone *get your finger out* is close to euphemistic dysphemism with its implication that the finger is in some unmentionable human orifice.

What Australians call *the bush dunny* is more figuratively called *the long drop* because there is indeed a long drop from seat to cesspit—one comparable with the drop from a garderobe in a medieval castle or a Jacobean manor house in Britain.

A woman’s breasts (but not the less salient man’s) are variously called *knockers, bouncers, bulbs, balloons, bazoom(b)as* (a remodelling of *bosoms*^28^), *globes, headlights, melons, montezumas* (remodelled blend of *mound* and *bosom*), *mounds, molehills*; *a pair, a set*; *lungs*. Perhaps the term *norts* is based on *noughts* capturing the outline of breasts such as depicted in rough two-dimensional sketches. Australian *norks* is perhaps partly derived from *norts* by deliberate remodelling or mishearing, but more likely from the trade name *Norco Cooperative Ltd*: it was a New South Wales butter company whose advertisements once featured a cow with an exceptionally large udder. *Jugs* has a similar associations with a milk container.

(19): Well, about five nights ago he caught me when I was drunk and horny and I ended up showing him how the cow ate the cabbage [how to have oral sex]
  (Burroughs 1985: 34)

Both *cauliflower* and *cabbage* are euphemisms for genitalia. The flaccid penis is a *tail* (the term *penis* is Latin for “tail”), a *tassel, bauble, putz* (this word is much less dysphemistic in English than in Yiddish; it derives from the verb *putsn* “adorn”) It is a *dangle, worm, schlong*, *tummy / hairy banana* (cf. Bahasa Malaysia *pisang* “banana”, said to be the prevalent euphemism used among women), *noodle, dill, gherkin, wally, wire, wiener* and other terms for sausages (*little boys sport saveloys*). One delightful metaphor is *the one eyed trouser snake*. *Cock*—which, in this sense, has multiple sources—comes in part from the “tap, faucet” sense of *cock* (still present in *stopcock* and *ballcock*) (see Allan and Burridge 1991: 105ff); *doodle* derives from *cock* via fore and end-clipped *cockadoodledoo* but also perhaps from *doodle* as adornment. The bird metaphor sanctions *pecker*. It is found in Catullus (85–54 bce) *Passer, deliciae meae puellae* “sparrow, my girl’s darling” (Carmina II, Catullus 1969) and throughout the centuries since. Used literally, *cock* is the generic term for a “male bird”; however, its salient literal meaning is “the male of *gallus domesticus*”; and this cock, by repute, rules the roost—whence *rooster*. This used to be what a man was expected to do (a view which probably had some effect on the use of *Cock* as a euphemistic remodelling of *God* in fifteenth–seventeenth century English). Taking this perspective, for a man to be *cock of the walk* demonstrates the very essence of manhood. From a different point of view, the manifestation of maleness is having a penis. From the Middle Ages until at least the seventeenth century, the flesh and blood of a cock was believed to be a strong therapeutic and restorative agent and was recommended in medical texts and recipes. Thirdly, there is the folk myth that men often find themselves sexually aroused at cock-crow (hence the wake-up call *Wakey, wakey, hands off snakey*), whereas their womenfolk are more readily sexually aroused in the evening. Because the penis rises with the cock (rooster), an association is established between the two. Fourthly, the rooster is a randy creature which struts around with a neck that moves not unlike an erect penis on a walking man, whence one source for the idiom *keep your pecker up*. *Pecker* is a frequent euphemism for *penis*. A *pecker* was also a narrow hoe used for digging holes when seeding; perhaps it got the name from the birdlike action involved in its use, and the association with seed. Certainly, peckers, seed, and birds form a natural set which intersects with another natural set whose members are peckers, penises, seed, and holes. *Beak* is one of the many synonyms for “penis” (Farmer and Henley 1890–1904). The similarity of the profiled penis-with-testicles to the outline of a bird gives rise to an image found in many languages, cf. the Bahasa Malaysia and Bahasa Indonesia euphemism *burung* “bird” (used particularly by women); Italian *uccello* “bird” and *passerotto* “sparrow”, Latin *passer* “sparrow’; and the German verb *vögeln* “fuck” is apparently based on *Vogel* “bird” (Barolsky 1978; de Jongh 1968/1969). The guy who said he was in prison ‘not for robbing a wagon but for wagging his robin’ was immediately understood (Mike Harding ‘Strangeways Hotel’ (Rubber Records, 1975). One of the appearance-based terms for the female pudendum is *bird’s nest*. The penis is the bird to enter this nest. Mark Twain wrote an imaginary conversation, (20), in mock Elizabethan English between Queen Elizabeth I, Lady Helen (aged 15), Lady Alice (aged 70) and Francis Beaumont (aged 16):

The ambiguity of *cock* in English is found elsewhere in Indo-European. Latin *gallus* had a meaning “penis” from classical times, through Vulgar Latin, and this meaning was maintained in Italian and Spanish. However, Latin and its daughters do not generally associate *gallus* with taps. French *coq* has many sexual associations: *coquille* (“shell, ornament in the shape of a bird’s beak, vagina”), *coqueter* (“copulate with a girl”), *coquer* (Lyon dialect)”‘kiss or embrace as the cock does hens”, *coquelier* (“run after young girls”), *coquine* (“prostitute, male homosexual”), *coquard* (“ridiculous old beau”), *coquardeau* (“male flirt”), and *coqueluche* (“ladies man”) (Baird 1981). There are also German *Hahn* which means or has meant all of “rooster, penis, spout / tap”; and Swedish *kuk* and Danish *kok* meaning both “rooster, penis”, but not “tap”. And finally there is the early fifteenth century English poem ‘I haue a gentil cook’, (21), which is contemporaneous with or slightly earlier than 1481, the earliest recorded use of *cock* in the sense “tap”.

(21): I haue a gentil cook, crowyt me the day;
  He doth me rysen erly my matyins for to say.
  I haue a gentil cook, comyn he is of gret;
  His comb is of reed corel, his tayil is of get.
  His leggis ben of asour so gentil and so smale,
  His sporis arn of syluer qwyt into the wortewale.
  His eynyn arn of cristal lokyn al in aumbyr,
  And euery nyht he perchit hym in myn ladyis chaumbyr. (Silverstein 1971)

This delightful lyric can, of course, be interpreted quite literally as a poem about a pet rooster; but there is no doubt that it allows for a lewd interpretation, too. A very loose translation is:

(22): [1] I have a fine cock that wakes me early every morning. [2] He gets big (or, He comes of good stock). His tip is coral red and his root is buried in black hair. [3] He has fine blue veins running up the side of him, like legs. When erect, he’s milky white underneath where he joins my scrotum. [4] His eye discharges spunk the colour of crystal, but more often amber-coloured piss. And every night he enters my lady’s quim

The gross rendition in (22) destroys the subtlety, wit, and beauty of the original. The interpretation of stanzas [3] and [4] is admittedly far-fetched, but justified as follows. This bawdy poem cloaks its bawdiness behind a source domain: the description of a rooster. The lyric is completely consistent with this description, and like other bawdy puns it relies on one or two salient aspects of the source domain to evoke the target domain, in this case a penis. The process is the construction of two parallel images: the image of a rooster and of a penis with the attributes of the rooster transferred to it when consistent. All understanding is a constructive process on the part of an audience; and with puns and metaphors, it is doubly constructive. Two people hearing the same literal statement may well put different constructions upon it (that phrase is telling!); two people interpreting the same metaphor have far more scope to create different constructions. When interpreting a poem like this one, the two different constructions can both be right.

The poem ‘I haue a gentil cook’ offers very strong circumstantial evidence that, in English, *cock* ‘penis’ is at least as old as *cock* “tap”. Given that *cock* in the tap sense was likely to have been written down soon after it became current, whereas the taboo sense was probably current long, long before it got written down, the taboo sense is very possibly older and part of the Indo-European heritage of English. Ashley Montagu writes:

- *God damn*, that favorite expression of the Englishman, though it is known to have been in common use at the beginning of the sixteenth century [for which Montagu has shown conclusive evidence] is not recorded in an English work until the end of that century. This clearly shows how much older are many of the oaths with which we have dealt and shall have to deal than the date of their first written or printed appearance. (Montagu 1968: 124)

There can be no doubt that taboo words are regularly much older than their first recorded use.

Whatever the historical truth, the reason that Americans and many Australians use *rooster* for *gallus domesticus* where the British still use *cock* is exactly because speakers readily correlate *cock* “bird” and *cock* “penis” today. Non-taboo homonyms are often abandoned in this way because people do not wish to (appear to) be offensive.

There are the bell-clapper metaphors for the penis: *ding, dink, dong, donger* (as in the Australian proverb *Dead as a dead dingo’s donger*). There are probably two sources for the bell-clapper metaphor. First, the vagina is seen as a bell, activated by the penis-clapper. The clitoris is sometimes called a *bell* at the entrance to the house of love (it activates the owner); yet this *love button* looks more like a bell-clapper. Second, the silhouette of *a man’s tackle* (what Americans might call his *basket*) is not unlike that of a bell, and the ungirded (flaccid) penis bouncing against the scrotum of a walking or running man is visually similar to the bell-clapper at work (even if it is external rather than internal). The basically onomatopoeic expression *tinkle* “urinate” may be reinforced by the bell-clapper metaphor.

The erect penis is likened to a weapon: *weapon, sword*, (*vagina* is Latin for “sheath, scabbard”), *pistol, gun, rod, lance, bill, pike, dart, chopper, prick*.^29^ The very common *prick* in the sense “penis” is arguably a literal sense rather than a nonliteral one. The verb *prick* names the effect of a certain kind of event in which a sharp object penetrates a membrane; with little stretching of the imagination that describes the effect of inserting the penis into the vagina: intromission is an act in which the penis is the instrument of pricking, i.e. a prick, which makes this noun deverbal. According to the *Oxford English Dictionary* the noun and verb have co-existed since the earliest records in English. In addition to its current meaning, the verb *prick* has meant “to spur or urge a horse on” (*OED* 9–12) which links up with the copulation-as-riding metaphor, with the man as rider; “to thrust a stick (or pointed object) *into* something” (*OED* 25) which links up with the metaphor of the man as *tailor stitching* the woman—the man as *tailor* turns up in many bawdy folk songs, and in Grose et al. (1811); and *prick up* still means “to rise or stand erect with the point directed upward” (*OED* 28). It is hardly surprising, then, that the noun *prick* was used variously for (a) a thorn, a sting, and figuratively as a vexation or torment (*OED* 12); this could be partly responsible for some interpretations nonliteral *prick*; (b) a dagger or pointed sword (*OED* 15) links with the penis as weapon metaphor; (c) the upright pole of a tent (1497, *OED* 16); (d) it has long been a term for the penis. Although the earliest record for *prick* “penis” in the *OED* is 1592, there is the record of it being used as a term for a lover in 1540 (*OED* 17b), which suggests at least a contemporary sense “penis”, too. In all probability, *prick* was used much earlier in this sense.

Even if *prick* “penis” was originally nonliteral, with the passing of time it has established for itself a separate identity. The original motivation for many other words is nonliteral too; yet they are now taken be literal: for example, the noun *crane* “lifting device” was based on its visible likeness to the bird; the *pupil* (of the eye) was, before being adopted from Latin, a metaphorical “child of the eye”, cf. *school pupil*; now both senses are taken to be literal. One difference between *prick* “penis” and the words *crane* and *pupil* is that *prick* seems closer to its nonliteral origin than *crane* and *pupil* to theirs.

Variants on the *prick* image are *needle, pin, thistle, hook, horn*, *bugle* (originally made from the horn of an ox)*, pencil,* and *pen*. These give rise to the metaphor of the *tailor* as the male partner and *stitching* for “copulating” in numerous folk songs; and the *doctor* giving his lover a shot with his *needle* in many blues songs. Other metaphors are *machine, instrument, tool, hammer, poker, pipe, knob, pole, shaft, staff, stand, oar, bone(r), hard, stiff* and [*get*] *wood*. There is also the once very common *yard*: Is this male fantasy or a variant of *hard*?

*Bollocks* or *ballocks* (the term invariably used by John Wilmot, 2nd Earl of Rochester, 1647–1680)^30^ derives from ‘little balls’, cf. *butt* ⇒ *buttocks, bull* ⇒ *bullock*. A link between *bollock* and *bullock* (Clemens 1968: 23) is feasible, given the long European tradition of bull-worship in which a bull’s testicles figured as a symbol of virility—but there is no textual evidence. Metaphors include: *balls, billiards, nuts, stones, rocks, marbles, pills,* etc. *Goolies* probably derives from Hindustani *goli* “pebble, ball, bullet” (McDonald 1988). The old established *cods* (whence *codpiece*) is from Old English *codd* “bag”.

(23) is part of *The Trooper watering his Nagg,* an early eighteenth century song which is another example of artistic euphemism, one that exploits appearance based metaphors.

(23): Quoth she what is this so stiff and warm,
  Sing trolly, lolly, lolly, lo;
  ’Tis Ball my Nag he will do you no harm,
  Ho, ho, won’t he so, won’t he so, won’t he so.
  But what is this hangs under his Chin,
  Sing trolly, etc.
  ’Tis the Bag he puts his Provender in,
  Ho, ho, it is so, etc.
  Quoth he what is this? Quoth she’tis a Well
  Sing trolly, etc.
  Where Ball your Nag may drink his fill,
  Ho, ho, may he so, etc.
  But what if my Nag should chance to slip in
  Sing trolly, etc.
  Then catch hold of the Grass that grows on the brim
  Ho, ho, must I so, etc.
  But what if the Grass should chance to fail,
  Sing trolly, etc.
  Shove him in by the Head, pull him out by the Tail,
  Ho, ho, must I so, etc. (D’Urfey 1791, Vol. 5: 13f)

Very commonly, the vulva is wrongly called *vagina*; the word derives from Latin *vulva* or *volva* “wrapper, uterus”. The vulva, vagina, and uterus were often not separately labelled—neither in English nor in many other languages—presumably because of their common generative function. There is a close link between *vulva* and *valve* from Latin *valva* “(leaf of) a folding door” and cover of the vagina of certain flowers, and hence applicable to human anatomy. But this is folk etymology. It was once supposed that exposing the vulva can defeat evil (see Allan and Burridge 2006: 7). This empowerment may be the point of fifth century bce Baubo figurines and the *Sheela*-*n*-*Gig* images found in medieval castles and churches displaying the vulva (Allan and Burridge 2006: 8). There is probably a link (though in which direction?) with the term fourteenth–twentieth century *gig* “loose woman” and hence “cunt” for much of that period.

Queen Elizabeth I (1533–1603) was probably unaware of these ancient beliefs in the power of the displayed vulva. It is, however, in keeping with her tough-minded instinct for survival that she (perhaps only apocryphally) chided her troublesome male courtiers ‘If I had been born crested not cloven, your Lordships would not treat me so’ (Rycroft 1979: 75). The vulva is seen as a *cleft, furrow, valley*. It is also described as a *boat* (the clitoris is *the man in the boat*), which refers to the configuration, but also has associations with water (and fish). *Twat* is possibly associated with *two*—*twa* because of the silhouette of the labia majora; the origin of the final excrescent-*t* (it is a long-shot to link it to *dyad*) is more mysterious than the final—*t* of *cunt* by which it was perhaps influenced.^31^ The terms *triangle, Y, pie* probably take in the pubic mound as well. There are several ancient carvings that justify the persistence of this image.

‘Focative’ is intended to evoke *firk* or *fuck*; *case* and *O* are euphemisms for “vagina” and “vulva” respectively. A *caret* is wedge-shaped, a shape traditionally associated with a woman’s pubic triangle, and there is also a play here on *carrot* “penis”. It has been said ‘The great cleft is called […] the cunnus, because it looks like the impress of a wedge (cuneus)’ (de Graaf 1672 quoted in Blackledge 2003: 87). This is folk-etymology. ‘[T]hat’s a good root’ has almost the same meaning in modern Australian slang that Shakespeare alludes to here. ‘’Oman’ is simply a play on the vocative ‘O man’—like Alice’s ‘O mouse’ in *Alice’s Adventures in Wonderland*.^32^ The vulva itself is also a *ring, circle, do(ugh)nut, dial, wheel*. Shortly after Zsa Zsa Gabor was convicted for slapping a cop who had arrested her for a traffic violation, the following caption appeared under a still photo from a commercial she was filming:

(25): ZSA ZSA’S GAME: The flamboyant Hungarian actress re-enacts her cop encounter in her new ‘Wheel of Fortune’ commercial for New York’s WCBS-TV. In one version, Gabor coos, ‘… take away my driver’s license. But darling, don’t touch my wheel.’ (*USA Today* Monday, October 23, 1989: 2D)

The vulva is also known as the *slit,*^33^*slot, crack* (hence *cracksman* for ‘penis’, and the dysphemistic *bit of crackling* for ‘woman’), *breach, gash,* (*everlasting*) *wound* (the link with menstrual blood). It is a *gate, hole, tunnel, den, box,* (*genitive*^34^) *case, hat* (see below).

*Cunny* “cunt”, spelled variously and retained in modern *cunnilingus*, derives from Latin *cunnus*, probably as a euphemism. There may also have been some input from French *con* itself derived from Latin *cunnus* and used for the bawdy-part from (at least) the fourteenth century (cf. Boch and von Wartburg 1975; Picoche 1979), and perhaps from Spanish *coño*, too. *Coney* /kʌni/ was the word for “rabbit” until the late nineteenth century, when it dropped out of use because of the taboo homonym. In Latin, rabbit is *cuniculus*, and its burrow *cuniculum*; end-clip either and you are left with *cuni[e]* (spelled variously as *coney, cony, conny, conye, conie, connie, conni, cuny, cunny, cunnie*^35^). One of the many euphemisms for *cunt* was *cunny*-*burrow*, hence the picturesque term for a penis as the *cunny*-*burrow ferret* (Farmer and Henley 1890–1904). There is a long-time link between rabbits, bunnies, and cunts. *Rabbit* is usually a term of abuse when ascribed to a woman (cf. Mitford 1979: 347). Playboy’s ‘bunnies’ and ‘Bunny Club’ followed a long tradition going back beyond eighteenth century London’s ‘Cunny House’ (Leach 1964: 50). Though the evidence is unclear, it may well be that *bunny* (which appeared in the late seventeenth century) was a euphemistic remodelling of *cunny*: it was a term for rabbits, rabbit tails, bony lumps on animals (reminiscent of the *mons veneris*) as well as an affectionate name for a woman. *Bunny* was also a dialect term for “an opening or ravine in a cliff”—which is suggestive. If the initial ‘b’ was indeed some sort of euphemistic remodelling device in the case of *bunny*, consider also not the baseball term, but the nautical term *bunt* “a cavity, pouch or bagging part of a sail or net; the funnel of an eel-trap”: is that also remodelled? Along with talk of women, rabbits were one of the few land animals that used to be tabooed by sea fishermen. *Bun* is listed by Grose et al. (1811) as ‘A common name for a rabbit, also for the monosyllable’ and it is still in use.^36^*Bun* was also the name for the tail of a hare (associations: hare~hair~pubis; tail~cunt) and for a squirrel: *squirrel* was one term for a prostitute; *bunter* was another. There is even a link between rabbits, hares and cats (pussies): Grose notes that *ma(u)lkin* or *mawkin* is a “cat or awkward woman” and in Scotland “a hare”; Baker (1943)^37^ lists “rabbit” as one meaning for *pussy* in Australian, though I know of no Australian who has heard this usage; the *OED* tells us *puss* meant, *inter alia*, “hare”. There is evidently a set of connexions between cunnies and bunnies and hares and pussies.

In Australia at least, a sexually active teenage girl was and is a *mole*. This is probably a pronunciation-based spelling for the homophonous *moll* but also partly sourced in the furry animal associated with burrows.

Though it may be excrescent, the final –*t* of *cunt* is probably inherited from Germanic: Old Norse *kunta* and Middle Low German *kunte*. Old Dutch *kunte* became Middle Dutch *cunte*, although in Modern Dutch it is *kutt*. In Middle English it was variously *kunte, cunte, counte, count, cunt*(*t*). The occasional homonymy with the Romance title *Count* may explain why the latter was dropped in favour of Saxon *Earl.*^38^

There may be a pre-historical association between *cunny / cunt* and *cu / ku* “cow” from Old to Middle English (plural *cy / kyn / kine*, among other spellings)—long an unflattering term for a woman. In some English dialects, between the thirteenth and the late nineteenth century, this body part term was homophonous with the adjective *quaint*—which, from the thirteenth–sixteenth centuries was also spelled *queynte* (among other ways), for instance by Chaucer in line 3276 of *The Miller’s Tale* and in the *Prologue of the Wyves Tale of Bathe*.

(26): For certeyn, olde dotard, by youre leve,
  Ye shul have queynte right ynough at eve. (331f)
  What eyeleth yow to grucche thus and grone?
  Is it for ye wolde have my queynte alone? (443f)

Later the Wife uses a euphemism when recounting a dalliance with a man half her age:

(27): I hadde the prente of seinte Venus seel
  As help me God, I was a lusty oon,
  And faire and riche, and yong, and wel bigon [“dressed”],
  And trewely, as myne housbondes [she is on her fifth at this point] tolde me,
  I hadde the beste quonyam [“whatsit”] myghte be. (604–8)

Florio 1611 in his Italian-English *Dictionarie* used the spelling we use today: ‘Fíca,… *Also used for a woman’s quaint*.’ In those far off days, the adjective *quaint / queynte* meant much the same as it does today but, if anything, it was more laudatory; Chaucer again:

(28): We wommen han, if that I shal nat lye,
  In this matere a queynte fantasye;
  Wayte! what thyng we may nat lightly have
  Ther-after wol we crie al day and crave. (*Prologue of the Wyves Tale of Bathe* 515–518)

If there is a phonological link between *cunt* and *quaint*, it may lie with labialized onset to Old English *cwiðe* “womb”, *cwene*, Middle English *que(y)ne*, Modern English *quean(e)*, a dysphemistic term for a “woman” that came to mean “whore”. Irish *cuinte* and French *coin / cointe* (“cunt”) also have labialized onsets; as does current English *quim*. This is partly sourced in *queme / quim “*something pleasurable, snug, intimate”; and partly perhaps in Welsh *cwm* “cleft, valley” although this latter is pronounced /kuːm/. The Old English counterpart is *cumb*, which occurs in place names like *Eastcomb* and *Cumbria*, and is cognate with Norman French *combe.*

*Well, bottle* and *pond* all mix configuration with function and/or effluvia in their imagery. The vulva is seen as a *mouth*, with lips and tongue (clitoris)—hence, *nether*-*lips*. Like the mouth it salivates and drinks, and can *flash an upright grin*. Such metaphors, like others for tabooed body parts, liken it to a non-taboo part. Terms like *bite, snatch, vice / vise, snapper, clam* and *oyster* extend the metaphor by suggesting a mouth ready to snap up a penis; the myth of *vagina dentata*—the vagina with teeth that may mutilate a man—is found in Africa, America, Europe, and India. *Vice / vise* “tool for gripping” is doubtless immorally inspired, too. Note that *snapper, clam* and *oyster* are also fishy—a fishy odour being commonly attributed to this organ; we therefore find terms like *fish(tail)* and *ling* for “vagina” (and *hook* for “penis”); *mermaid* was a euphemism for “whore”. The plant *Chenopodium vulvaria,* also known as stinking goosefoot ‘readily told by its repulsive smell of decaying fish’ (Fitter 1971). The noun and verb *fishfinger* denote “digital stimulation of a woman”; and *fishing* or *angling* “digital stimulation of the vagina; copulation”, and *fishbreath* arises from “oral sex” (Allen 1987). Grose et al. (1811) list the wonderful metaphor *the miraculous pitcher, that holds water with the mouth downwards*: it seems unlikely that this lengthy example of verbal play was widely used, and its flippancy is reminiscent of euphemisms like *kick the bucket*^39^ for “die” with their real or pretended disdain for a taboo.

As always, Shakespeare offers some witty material:

The images here include: (a) A man over a woman; (b) the woman keeping her private parts hidden (‘below stairs’); (c) a woman as mouth; (d) a man’s foil which scores a hit but does not hurt; (e) a buckler is a small shield with a boss to ward off thrusts from daggers, swords, and pikes; a maid’s buckler is the boss of her mons veneris (“mound of Venus”, note the metaphor in this term, described in a dictionary of 1693 as ‘the upper part of a Womans Secrets, something higher than the rest’); (f) a woman’s vagina between her open legs forms a vice (vise) in which to put the pike; (g) if swords and pikes are penises they are indeed dangerous to maidenhead.

To *have someone by the short and curlies* makes implicit reference to pubic hair; it is a euphemistic and colloquial counterpart to the dysphemistic *have someone by the balls*. Pubic hair on the mons veneris, *the grass on fanny’s hill*, is both visually and tactually salient, not to mention erotic; not for nothing was pubic hair airbrushed out of soft-porn photographs until the 1960s. Many women remove its periphery (*trim the borders*) so that it does not violate taboo by poking out of skimpy briefs, but also to look more little-girl-like. *Vag* is end-clipped from *vagina* gives rise to *vajay*-*jay* whose reduplicated suffix is an affectionate diminutive. Actress Jennifer Love Hewitt says (http://www.youtube.com/watch?v=NvzhvKm_15k) ‘Women should vajazzle their vajay-jays’ in other words dress up a Brazilian wax (which removes all or virtually all pubic hair) with crystals applied to the pubis. A first century graffito from Pompeii notes the fact: futuiturcunnus [pil]ossusmultomelius [qu]amglaber “a hairy cunt is much better to fuck than a hairless one”(see Read 1977: 22). One of the appearance-based terms for the female pudendum is *bird’s nest*. The penis is the bird to enter this nest—as we saw in (20). In contrast to many men, on most women pubic hair is the only substantial patch of body hair.

(30): When you’re standing up, all you see from the front is hair. Between your legs there are two soft, cushiony things, also covered with hair, which press together when you’re standing, so you can’t see what’s inside. (Anne M Frank, aged 15, March 24, 1944; quoted in Blackledge 2003: 55; cf. Frank 1997)

Its salience is clearly demonstrated in art, for instance in René Magritte’s abstract painting ‘Trois femmes’ of 1922, his ‘La ruse symmetrique’ of 1928, or Stella Bowen’s ‘Reclining nude’ of 1928. It is less picturesquely recognized in the following graffito from a woman’s toilet wall in Melbourne in 1988.

(31): The cunt is a mythical creature
  All matted and covered with hair
  It looks like the face of my teacher
  And smells like the arse of a bear.

The earliest uses of *merkin* seem to refer to the female pudendum as well as a *cunt rug* or *intimate wig* that may also sport a false vulva. The significance of pubic hair on the mons veneris accounts for the several furry animal terms *cunny,bun[ny], beaver,pussy* and, of course, *hare* (homophonous with *hair*).

(32): The picture Garp looked at in the dream was considered among the highest in the rankings of pornographic pictures. Among pictures of naked women, there were names for how much you could see. If you could see the pubic hair, but not the sex parts, that was called a bush shot—or just a bush. If you could see the sex parts, which were sometimes partially hidden by the hair, that was a beaver; a beaver was better than just a bush; a beaver was the whole thing: the hair and the parts. If the parts were *open*, that was called a split beaver. And if the whole thing *glistened*, that was the best of all, in the world of pornography: that was a wet, split beaver. The wetness implied that the woman was not only naked and exposed and open, but she was *ready*. (Irving 1979: 318. Emphasis in original.)

In addition, a *beaver* was a once a kind of hat; so called because it was made from felted beaver fur. A hat is a concave object into which a man puts his head; and the *glans penis* (note the Latin) is often referred to as its head. Hence Grose’s ‘Hat. Old hat; a woman’s privities: because frequently felt.’ In medieval Europe, beaver oil was regarded as an aphrodisiac.

There are also: *the hairy chequebook* (there are other many figures which treat genitalia—but especially female genitalia—as a source or store of wealth: *(family) treasure, treasury, crown jewels*—usually reserved for males*, jewel, money, moneybox*); *beard, bearded clam, hair pie, hairyfordshire* (play on the English county name *Herefordshire*)*, bird’s nest, cuckoo’s nest, cunt curtain, cunt down, mossy mound, park, grass* (‘On her belly there is a sign, Keep off the grass, the hole is mine.’), *ling* (“heather” not the fish), *furry mound, muff* (men wore muffs in times past, as women still do; muffs were usually made from fur and hands were put in them; however, one source for this word may be a remodelling of *mouth*—which is part of the food-eating metaphor for sex), *velvet, thatch, thicket, bush, brush, fuzz, pelt, pubes,* etc. One term for a woman is a *furburger* (the eating metaphor again). Not surprisingly, one term for penis is *hairsplitter* and copulation is *a poke in the whiskers*.

The link between women and cats extends way back. The Egyptian goddess Bastet had a cat’s head on a woman’s body and thousands of cats were mummified, at least partly in her honour. Grose et al. (1811) list in sequence ‘Tib. A young lass. Tibby. A cat.’ Japanese brothels are marked by the sign of a cat. French terms for vagina are *le chat* “the cat” and more especially *la chatte* “the female cat”—a term politely avoided in modern French. In Italy the words for female cat, *chatte* and *gatta* also mean “vagina”.

(33): **puss** (pʊs), *n.*1 Also 6–7 **pus**, **pusse**. [A word common to several Teutonic langs., usually as a call-name for the cat (rarely becoming as in Eng. a synonym of ‘cat’): cf. Du. *poes*, LG. *puus*, *puus*-*katte*, *puus*-*man*, Sw. dial. *pus*, *katte*-*pus*, Norw. *puse*, *puus*; also, Lith. *puźpuiź*, Ir. and Gael. *pus*. Etymology unknown: perh. originally merely a call to attract a cat.] (*Oxford English Dictionary*)

English *pusse* is first recorded referring to vulva and/or vagina in the seventeenth century (though it may well have been in use earlier). We can be certain that *pussy* in this sense has become established through a network of interacting associations. (a) The salience of pubic hair on the human body leads to the names of several furry animals (*beaver, bunny, pussy, ferret, rat*) becoming slang terms for the genitalia of women and men. (b) There is the link between rabbits (coneys), hares, and cats. (c) There is the long tradition of likening women to cats (men to dogs, ferrets, rats, cocks). As an endearment, a woman is likened to a *puss* and a kitten (*sex*-*kitten* perhaps); hence *kitty*. (d) The *OED* lists *pusse* and *pus* used disparagingly of a woman from the early seventeenth century. From the sixteenth century, *Kit* was a typical pseudonym for a prostitute and became a term for female genitalia, giving rise to the euphemism *kit has lost her key* for “menstruate”. (e) A *kitty*, like a *purse*, serves as a source or store of money. (f) *Purse, burse* was a “money bag” (drawn together at the opening) but extended to a “natural receptacle in an animal or plant”. Thus Medieval Latin *bursa* “bag, purse” was used of both scrotum and womb. The opening of a purse resembles a vulva; consequently Japanese *isoginchaku* “sea purse, *actinia malacoterem*, a kind of sea anemone”, also has the senses “round coin purse (which when squeezed opens the slit)” and “vagina” (Solt 1982: 78); furthermore, purses hold money, and a number of euphemisms for the vagina recognize it as a source or store of wealth. *Purse* has long been used as a euphemism for female genitalia, e.g. ‘The whores factors would faine drawe customers to her burse of bawderies’ (1617).^40^ A more recent example is:

The vagina is a *purse* partly because it is seen as a source or store of wealth; and partly because of the visual resemblance between a slit-top coin purse and the vulva and also lips: cf. *purse the lips*. In this context, there is possible influence from Irish *pus* ‘lips, mouth’; cf. modern English slang *puss* for ‘face’. (g) The earliest recorded form of *pussy* in the sense under discussion was in fact *puss*, and this is a spelling pronunciation for *purse* in some English dialects. Compare these more or less synonymous doublets: those on the left are colloquial with a short lax vowel where the standard counterpart on the right has a long tense vowel.^41^*Ass*~*arse; bass*~*barse; bin*~*been; bubby*~*baby; buss*~*burse* [“purse”]; *bust*~*burst; crick*~*creek; critter*~*creature; cuss*~*curse; gal*~*girl; hoss*~*horse; hussy*~*housewife; mot*~*mort* [“young woman”]; *sassy*~*saucy; tit*~*teat; whids*~*words; wud*~*world*. It is certain that *puss*–*purse* is one of these doublets.^42^

**Fig. 1:**
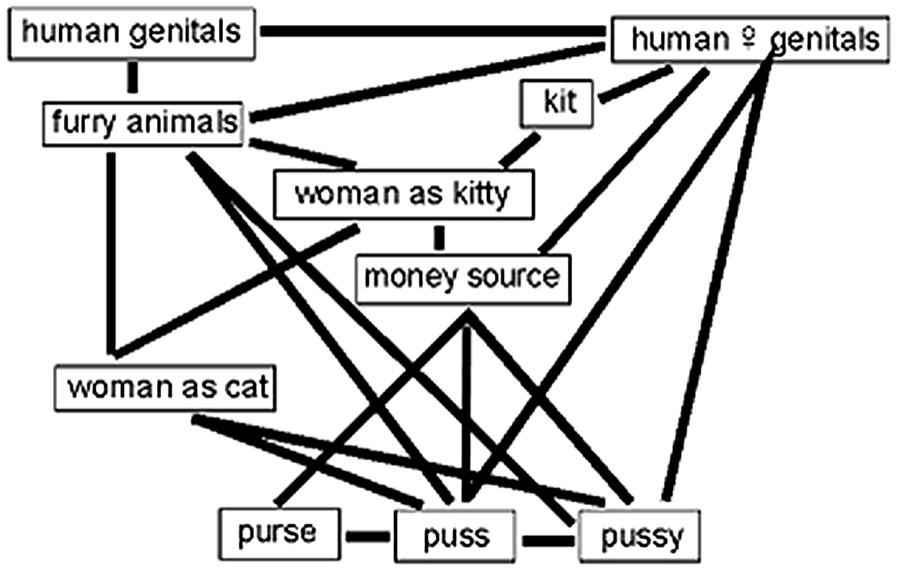
The *puss(y)* network of diachronic semantic relations

Figure 1 shows the interacting associations I have described falling together to motivate, inform, and confirm the meaning of *puss* and its endearment variant, *pussy*. Three semantic processes are found: (a) Metaphorical extension of *puss / purse* to the human female sex organ; (b) the kind of lexical confusion found in folk etymology: of *puss*~*purse* with *puss* ‘cat’ on the basis of semantic networks that associate women with cats, and furry animals with genitalia; (c) this has led to the semantic transfer of modern *pussy* to these networks and a severance of its historical relation to *purse*.

In the preceding discussion we have seen that (1) the X-phemisms created to talk about taboo topics give rise to language change through figures and metaphors based on physical appearance, function, and association; (2) metaphorical extension and lexical confusion contribute new meanings to old forms; (3) a new vocabulary item may have multiple sources. Although phonology and semantics are important constituents of this process, they are engineered and directed by pragmatics.

## The five senses

Allan (2009) investigated the connotations of English colour terms with particular attention to figurative uses of *black, white, grey, brown, yellow, red, green, blue* and a few miscellaneous colours. The connotations are judged on the basis of whether the phrases in which the colour terms occur are typically orthophemistic, euphemistic, or dysphemistic. All the colours surveyed have some, often many, orthophemistic connotations; euphemistic connotations of colours are rare; but dysphemism is common. *Black* is used orthophemistically but not euphemistically; it has dysphemistic connotations more often than other colours do. It is often connected to darkness (the night), death, decay, and evil deeds. *Black* has often been used dysphemistically of human skin colour though, like all racial dysphemisms and many other insults, it can be reclaimed as a badge of honour, and it can also be orthophemistic. *White* is in contrast to black and, as such, linked to light and purity; it mostly has positive connotations, though it is rarely used euphemistically. Dysphemistic uses depict cowardice and fear. Located on the achromatic scale between black and white, *grey* is, of course, used for indeterminability and dullness. It gives rise to few figures. The faecal associations of *brown* lead to several dysphemisms; *brown* is found in no euphemisms and few orthophemisms in figurative speech. In figurative expressions, *yellow* is dysphemistically used of cowards and cheap paper, and sometimes of East Asiatic people; but it is orthophemistic and positively used of light-coloured African-Americans. The occurrence of *red* in both positive and negative figurative expressions, links it with blood—life-blood, the blood of the slain, or menstrual blood. The colour *green* is linked to living vegetation; negative connotations arise when it is the colour of illness or jealousy (perhaps seen as illness). As the colour of the Madonna’s robe, *blue* is connected with the virtuousness and chastity of a bride. The negative aspects of figurative uses of *blue* arise from fear, fighting, despondency, and tabooed language and behaviour. It is arguable that the use of *blue* to speak about these topics is euphemistic and that uses of *blue* are rarely dysphemistic. Colour terms such as *gold, silver*, and *platinum* derive from the names for valuable metals from which they derive their mostly positive connotations. Other colour X-phemisms include the dysphemistic *purple prose* for language characterized by hyperbole and an overabundance of adjectives. The euphemism *be in the pink* means “to be in excellent health”; but *seeing pink elephants* is a playful if somewhat dysphemistic reference to inebriation. *Lavender linguistics* (about how lesbians, gay men, bisexuals, transgendered persons and queers use language in everyday life, and how language gets used against them by others) is certainly figurative but seems to me more orthophemistic than euphemistic. Products such as paints and lipsticks, sold principally for their colour, could be identified on colour charts by an alphanumeric code, but this doesn’t satisfy customer needs; instead, they are given names, many of which don’t even suggest a particular colour; instead, they are evocative of life style (e.g. for lipsticks *Belle, Berry Sexy, Day Dream, Soft Mocha, Strut,* and *Venus*; for paints *Nolita, Thomas Tallis, Lewis, Knowing, Highgate*, and *Sky Painting.*

All figurative uses of colour terms surveyed were, perhaps predictably, directly or indirectly based upon the visual attributes of the denotatum. Although individuals may experience synesthesia when encountering colour terms, the language resources demonstrate none. My attempt to classify the connotations of English colour terms reveals networks of associations, but no surprises. Presumably the kinds of process that lead to colour terms being used X-phemistically are universal, and it would be interesting to learn the extent to which the particular kinds of X-phemism recur in different languages.

(35): If you sprinkle
  When you tinkle,
  Kindly wipe the floor.
  (Notice in a men’s toilet, MASDAR House, Finchampstead, England 1989)
  If you sprinkle when you tinkle,
  Please be neat and wipe the seat. (Graffito in a women’s toilet, New York City, 1990)

The use of *tinkle* as a euphemism for *urinate* refers to the sound as the piss flows into water; it dates from after the invention of the water closet. The usually dysphemistic word *piss* is itself onomatopoeic and dates from time immemorial. It is possible that *piddle* is partly motivated by onomatopoeia, partly a remodelling of *piss*, and partly (for males) in imitation of a bird’s beak *piddling*, that is pecking for food as in the *OED* quote ‘sit at table and see a man pidling at his meat, picking and chusing’. It is probable that [*have a*] *slash* is onomatopoeic; though when a male urinates he is holding his weapon and if in the open, the line of piss may create a visual line like a slash through the vegetation. Other lavatorial onomatopoeia include the noun *fart* “break wind”, which is mildly dysphemistic, and the childish *plop*–*plop* “defecate”. There is sound symbolism of a different kind in referring to a lavatory as a *thunderbox*: this might have been inspired by the noise of toilet flushing and/or the noises made prior.

It is orthophemistic to speak of the *sense of smell* but to say of someone *You smell* is dysphemistic; in contrast *You smell nice* is a compliment (unless sarcastic). Pleasant smells are *fragrant* and *perfumed*. There are *perfumes* for women but *fragrances* for men and women. Like *smell*, the word *odour* has negative connotations; but whereas *nice smell* is an acceptable collocation, ??*nice odour* seems unacceptable. A *pong* and a *stink* and a *stench* have the same dysphemistic connotations of a bad smell. *Reek* has dysphemistic connotations: to say someone *reeks of perfume* means that the perfume is unpleasantly strong and *You reek* means the same as *You stink*. The fact that shit stinks gives rise to it being referred to as *poo(h)* which is a euphemism or perhaps euphemistic dysphemism. *Poo(h)* is related to *poof, phew, pho(ugh),* blowing through the lips (a very long voiceless bilabial fricative, in phonetic script [ɸɸɸ]) and similar onomatopoeic mimes for blowing something away—here the stench of shit. Related is *poo(h)* ‘an ejaculation expressing impatience, or contemptuous disdain or disregard’; for instance, when Ophelia speaks of Hamlet to Polonius ‘He hath, my lord, of late made many tenders Of his affection to me’ Polonius responds ‘Affection, pooh! You speak like a green girl’ (Shakespeare *Hamlet* I.iii.98ff).

*Sweetie, sweetness*, *sugar* and *honey* are all terms of endearment because human beings mostly like sweet tastes. As vocatives they are euphemistic alternatives to a name provided that the context is such that recipient can accept the appellation as sincere and appropriate from the mouth of the speaker. Appropriacy is dependent on acceptance of social closeness between the interlocutors at the time of utterance. Under other circumstances these terms become dysphemistic as sarcasm or unwanted over-familiarity. *Sour* is treated as the opposite of *sweet*. Although humans do eat and drink *sour tasting* things like acidic lemons and limes, foods that *turn sour* have gone bad and this is a dysphemistic attribution. *Sour breath* is bad breath (halitosis). To describe a person as *sour* or a *sourpuss* is certainly dysphemistic because it implies that they are “peevish, disagreeable, ill-tempered, of unpleasant character” given to experiencing *sour grapes*. *Bitter* is very similar to *sour*. Although certain bitter substances are ingested and English pubs sell *bitter* (a kind of beer which is not in fact very bitter), by and large *bitter* is at best orthophemistic in describing the taste of wormwood, quinine and the like, but usually dysphemistic in *bitter pill, the bitter end, a bitter wind, bitter words*. To describe a person as *bitter* implies that they are “afflicted by ill-feeling, unquenchable hurt or despair, and perhaps desirous of vengeance”. A *saucy* person is impertinent; but *saucy language* is hard to distinguish from *salty language* and even from *fruity language*—all of which may be found in a *spicy novel* (which will probably not have a *pithy* plot).

*Hot* is rarely used dysphemistically. A *hot property* is one highly regarded and desirable and therefore valuable (whether or not this is real estate or used of a starlet) of which one might exclaim *Hot dog!* Most figurative uses of *hot* derive from its literal use as something which generates heat and/or retains applied heat, as in the implied reference to blacksmithing in *strike while the iron is hot*—because the hot metal is malleable; hence ‘act when conditions are right’. Vigorous activity generates heat, hence the orthophemistic *hot rod* and *hot licks* in *hot jazz* and the euphemisms for sexual passion aroused in *have the hots for that girl, she’s hot stuff*. Most likely dysphemistic is *I have a hot cock for her hot pants*. It is possible that the rhyming Australian idiom *hot to trot* “ready and eager to get underway” is associated with sexual preparedness or more mundanely with the heat of a revved engine. It is usually at least mildly dysphemistic to describe someone as *hot blooded* “easily angered”, *hot under the collar*, or just plain *hot* “angry”. Because hot things burn, *the hot seat* was slang for the electric chair; however, the hyperbole *to be in the hot seat* often has much less fatal consequences in everyday use. To refer to stolen property as *hot* presumably derives from the risk of being figuratively *burned* if one is caught with it and prosecuted.

To describe someone as *warm*-*hearted* or just plain *warm* (in reference to their character) is complimentary. To be *lukewarm* about something is to be unenthusiastic about it. Describing a person as *cold* or *cold*-*hearted* “void of emotion” is dysphemistic. Interestingly, *to be cool headed* is complimentary, as is the current slang *(That’s) cool*; *to be cool towards someone* is usually mildly dysphemistic. *Chill (out)!* seems mildly dysphemistic when it means “Calm down!” but orthophemistic and positive when it means *Stay cool*.

*Soft skin* may be desirable in a child or a woman, but to describe someone as *soft* is a mildly dysphemistic, because it means “stupid”. Just as dysphemistic is describing someone as *soft on* drugs or crime. To describe someone as *hard* “unyielding, inflexible” tends to the dysphemistic; though to be *hard*-*headed* is to be “resolute, sagacious” and is a positive evaluation. To describe something as *smooth sailing* is to say that things went well and were easily accomplished and there are no negative connotations. And although the description *smooth operator* can be uttered in envy so that the person so described will not necessarily take offence, it has rather dysphemistic connotations. It is certainly dysphemistic to describe someone as *a slippery customer* or *slippery as an eel* which ascribe “deceit, shiftiness, unreliability”. *To be on the slippery slope* is to be approaching disaster.

*Rough* is more or less the opposite of smooth. A *rough draft* is unfinished, not polished. A *rough journey* and *rough time* characterize unpleasant events. *Rough words* are disturbing to the hearer and so have a dysphemistic effect. To describe someone as *rough* “not refined” is usually dysphemistic, however this is not really the case with someone described as a *rough diamond*; although it picks up mild dysphemism from the epithet *rough* this is coerced by *diamond* which is something tough and of great value. Consequently to describe someone as *a rough diamond* is complimentary—or at worst a dysphemistic euphemism. However, *to cut up rough* is an entirely negative assessment. If it is said of a woman that *she likes a bit of rough* it insults her choice of partner as being of a lower socio-economic class than she is.

(36): What do you mean the unions won’t like it, Jim? Don’t be so wet
  (Margaret Thatcher, quoted in the London *Observer* July 26, 1981 p. 3)

Thatcher was using *wet* dysphemistically to mean “soppy, ineffectual, lacking resolve”. Mostly, *wet* and *dry* and are orthophemistic. *Dry* is more often dysphemistic—or at least has more negative connotations—than *wet*: to be *dry mouthed* implies fear or thirst; *dry eyed* tends to be used when tears are expected, so implies hardness of heart; although *dry humour* is barely negative, it does suggest a lack of strong emotion and somewhat caustic wit; *dry facts* are unexciting; *dry bread* lacks the adornment of tasty butter and jam; a *dry cow* yields no milk; a *dry joint* makes a bad (electrical) connection through faulty soldering; a *dry fuck* is either one where no orgasm is achieved (by either party), or the woman is (physically) sexually unreceptive. As Janet says in the *Rocky Horror Picture Show*, heavy petting leads to *seat wetting* (from sexual excitement) (http://www.youtube.com/watch?v=z2na49WxKmo); its antithesis leads to the vile simile *dry as a nun’s nasty*. The dysphemism *dried up old crone* has nothing to do with sexual arousal but instead with the desiccated look that accompanies old old-age. Perhaps the only time that *dry* normally has a more positive spin than *wet* is where a *dry blow* is one that does not draw blood, whereas a *wet operation* is one aimed at killing. New Zealand slang *get* (*someone*) *wet* “gain the upper hand over someone” presumably comes from this bloody sense of *wet*. The American dysphemism *wetback* refers to illegal immigrants, originally those who crossed the Rio Grande from Mexico. Finally there is *wet behind the ears* “immature, naïve”; whether this was inspired by a new born infant as it emerges from the birth canal, I do not know.

## Artful X-phemisms

X-phemisms, particularly—but not solely—those for the activities of and effluvia from tabooed bodily organs often exhibit verbal play. I begin with a few lines from the world’s greatest master of double-meaning, William Shakespeare. Almost the whole of Act II scene iii of *Romeo and Juliet* is packed with euphemisms whose ostensible meaning would not trouble the prudish Thomas Bowdlers of this world, but whose innuendo would certainly not have been lost upon the Elizabethan or Jacobean audience. There are three young men talking smuttily, as young men will; then Juliet’s Nurse arrives on the scene looking for Romeo, whom she doesn’t know, and Mercutio jests at her expense.

I draw attention to the following phrases and offer the occasional gloss and commentary but leave the rest to the reader.

- groaning for love
- this drivelling love is like a great natural [either a whore or a simpleton]
- hide his bauble [penis] in a hole
- my tale [homophonous *tail* is slang for “penis”] against the hair [female pudendum]
- have made thy tale large
- I was come to the whole depth of my tale; and meant, indeed, to occupy [copulate with] the argument no longer
- goodly gear [genitalia]
- A sail! a sail! a sail! [assail (chat up) this woman]
- A shirt and a smock [woman (a smock is something one slipped into)]
- Anon [perhaps a gratuitous play on *a nun* “a prostitute”]
- her fan’s [fanny, female pudendum] the fairer face
- God ye good den [fanny, cf. *box*]
- the bawdy hand of the dial [female pudendum and owner thereof] is now upon the prick of noon [when the pointer is upright]

It is worth noting that the Nurse does twig what Mercutio is doing, because she says of him after he is gone: ‘Scurvy knave! I am none of his flirt-gills; I am none of his skains-mates’. Today’s equivalent might be ‘Bastard! I’m no tart; I’m not shagging him.’

There are many more examples in Shakespeare’s plays of such artful euphemism. Allan and Burridge (1991: 214–219) analysed in detail *Henry IV Pt2*, II.iv.22–125. Almost the whole of *Much Ado About Nothing* III.iv is a study in risqué girl-talk which suggests that well brought up women in Shakespeare’s day were far less coy about exchanging banter on sexual topics than their sisters of the eighteenth–twentieth centuries. *Twelfth Night* has Olivia’s puritanical yet vain and pretentious steward Malvolio left a letter by her maid Maria to gull him into believing that Olivia fancies him; Sir Andrew is one of the hidden onlookers watching Malvolio make a fool of himself.

Shakespeare relies on at least part of the audience seeing through the artful euphemism to recognize the sequence *c* + *u* +*’n’* + *t* (repeated in case the audience missed it) links up with a woman making *pee*—though this is never acknowledged by any of the characters in the play. It is a gratuitous dirty joke by the master of finely-honed wit. Small wonder that Shakespeare’s plays have an even larger audience today than they did when he wrote them.

Writers are well aware of the tingles provided by the artful euphemism. The author protects him or herself when talking about taboo topics by artfully trading on metaphor and figurative interpretations of the locutions used. There is a cline among such euphemisms that stretches from street slang to poetic allegories like the thirteenth century *Le Roman de la Rose* in which the lover’s quest to pluck the rose from the enchanted garden is an allegory of the pursuit of the flower of womanhood. The rose is a euphemism and symbol for the blood from a newly split hymen, a precursor to the more mundane account in *Memoirs of a Woman of Pleasure*, where Fanny Hill writes of her ‘virgin flower’ and of another girl’s ‘richest flower’ being ‘cropped’ with graphic descriptions of the bloody result (Cleland 1985: 77, 143). A bawdy interpretation of *Le Roman de la Rose* must be a construction in the reader’s mind, and that is where the art lies and the lie is artful. Queen Victoria’s wedding night was variously described in terms of a military advance, an exploratory foray, and an essay on horse-riding (Pearsall 1969). Works such as Jonathan Swift’s *Gulliver’s Travels,Tale of a Tub* and *Modest Proposal* (that Ireland’s overpopulation and poverty would be alleviated if the babies of poor Irish parents were sold as delicacies to be eaten by the rich) and George Orwell’s *Animal Farm* and *Nineteen Eighty*-*Four* are political allegories (Orwell 1946, 1987; Swift 1958). Shakespeare’s sonnets are, mostly, lyrical allegories, but his low comedy is closer (at least in spirit) to today’s street language. Through these artful analogies the writer achieves the heightened perception that an effective double-entendre will give and conceals just enough as to become all the more provocative and alluring.

As I said earlier, verbal play is not solely the prerogative of the skilled writer. Much longer lists than the following can be found on the web.

(39): Ambulance drivers come quicker. Astronomers do it with Uranus. Bankers do it with interest (penalty for early withdrawal!). Basketball players score more often. Beer drinkers get more head. Bricklayers lay all day. Chiropractors do it by manipulation. Crane operators have swinging balls. Dentists do it in your mouth. Detectives do it under cover. Doctors do it with patience. Fishermen are proud of their rods. Furriers appreciate good beaver. Gas station attendants pump all day. Golfers do it in 18 holes. Hairdressers give the best blow jobs. Handymen like good screws. Librarians do it quietly. Millionaires pay to have it done. Ministers do it on Sundays. Models do it in any position. Modem manufacturers do it with all sorts of characters. Professors do it by the book. Sailors like to be blown. Secretaries do it from 9 to 5. Stewardesses do it in the air. Taxidermists mount anything. Tennis players have fuzzy balls

All these sentences have an orthophemistic literal meaning and a sexually suggestive second meaning. Some people will avoid using a word which even sounds similar to a taboo term but there are others who will deliberately use such words humorously, as a tease; which is what the sentences in (39) achieve. A classic example is in *Monty Python’s Life of Brian*^43^:

There are the anal jokes found among boy scouts and soldiers, e.g. *Shit on a shingle* “chipped beef on toast”, *shit on snow* and *shit on lice* “gravy on boiled rice”; *scours* (a term for diarrhoea in livestock) “banana pudding”; *scoots* (“brown stains on underwear”, also known as *skid marks*) “chocolate pudding”; *scrotum* + *oatmeal* was the source for *scroatmeal* “oatmeal”; the drink Mountain Dew was the source for *mountain doo* “diarrhoea” also known as *Hershey squirts* (after Hershey chocolate) (Jay 1992: 27 possibly based on Mechling 1984). I know of a linguistics professor who gently taunts his graduate students by referring to the material gathered for their theses as *thecal matter*. And there is another who, while lecturing on the relationship of allophones to phonemes, inadvertently said ‘What the allophones of an abstract phoneme /A/ have in common, is their A-ness /ˈeinəs/.’ Having said it, he immediately recognized the ambiguity of the focussed term ‘A-ness’, so quick as a flash he asked, ‘And what do allophones of phoneme /P/ have in common?’ Cheris Kramerae and Paula Treichler suggested that their *Feminist Dictionary* should perhaps be called a ‘Cuntionary’. Cornog (1986) found that pet names used by (longer term) sexual partners for erotic body parts fall into variations on the owner’s name (*Little Willy* for Bill’s penis, *little Joanie* for Joan’s vagina); personal names (*Myra* and *Myrtle* for breasts, *Miss Muff* for the vagina, *Peter* for a penis); descriptive names (*Sweat Pea* for the clitoris, *Hot and Juicy* for the vagina); humorous names (*Omar the Tentmaker* for the erect penis under the sheets; *Ping* and *Pong* for testicles).

Euphemism often has a limited life expectancy; so there is a chronological turnover: for instance, today *sard* and *swive* have dropped out of use, their places taken by *bonk* and *shag*. Dysphemisms can be taken up as a means of empowerment, and so change status: this has happened with *gay* and, in Australia, with *wog* (“non-Anglo-Celtic white Australian”) and when *nigger* is used in the spirit of camaraderie among African-Americans (Allan 2015b; Kennedy 2003). Today’s PC terms are likely to suffer from the same historical recycling.

## Concluding remarks

X-phemism motivates language change by promoting new expressions, or new meanings for old expressions, and causing some existing vocabulary to be abandoned. In this essay we have seen that there are basically two ways in which X-phemisms are created: by a changed form for the word or expression and by figurative language that results from the perceived characteristics of the denotatum. Both processes, but particularly the latter, are pragmatically controlled. X-phemisms are motivated by a speaker’s want to be seen to take a certain stance and by playfulness.

Many X-phemisms are figurative; many have been or are causing semantic change; some show remarkable inventiveness of either figure or form; and some are indubitably playful. Euphemism, for instance, can be achieved antithetically by both hyperbole and understatement, by the use of learned terms or technical jargon instead of common terms, and conversely by the use of colloquial instead of formal terms, by both general-for-specific substitution and part-for-whole substitution, by both circumlocution and abbreviation, acronym, alphabetism or even complete omission, as well as by one-for-one substitution from the existing resources of the language or by borrowing from another language.

Dysphemism employs most of the same strategies as euphemism, but there are two main differences. One is that part-for-whole dysphemisms are far more frequent than general-for-specific ones, which is the converse of the situation with euphemisms: e.g. the use of *tits* for breasts^44^ is part-for-whole, as are figurative epithets like in *He’s a prick* which contrast with euphemistic counterparts showing whole-for-part substitutions like *chest* (speaking of a woman’s breasts) and (legal) *person* (referring to genitalia). Other differences between the strategies for euphemism and those for dysphemism are predictable: circumlocution is most usually dysphemistic when it manifests an unwanted jargon; the use of borrowed terms and technical jargon is only dysphemistic when intended to obfuscate or offend the audience; and so forth.

Euphemism as a work of art falls into three categories: there are the artful euphemisms, like many of those used in street language, which make a striking figure, but which are the everyday vocabulary of a particular jargon; there are the artful euphemisms which mask their original taboo denotations to such an extent that the latter are not generally recognized; and finally there are the artful euphemisms which are meant to be as revealing—and in their own way as provoking—as diaphanous lingerie. As bawdy authors like Shakespeare and political satirists like Swift and Orwell well know, titillation of the audience is the best way to draw attention to their message.

X-phemisms of all kinds display folk-culture, and arise through similar linguistic stratagems to achieve different effects. An interesting perspective on the human psyche is to be gained from the study of language expressions used as a shield against the disapprobation of our fellows or malign fate, and others used as a weapon against those we dislike or as a release valve against the vicissitudes of life. Many euphemisms and dysphemisms demonstrate the poetic inventiveness of ordinary people: they reveal a folk culture that has been paid too little attention by lexicographers, linguists, literaticians, and pragmaticists.

## Footnotes

1 Such salience is doubtless the motivation for this highly successful logo.

2 ‘I really don’t know what to make of these,’ said Norman as he sorted through his Christmas post. ‘It seems what I get more letters and parcels than what I know people, it surprises me more and more each year, as more of these parcels keep coming. I really don’t know who-all they bloody are—sending me all these.’

3 The adverb (?) *under* is converted into a plural nominal (like *pants*). The /ız/ suffix may be influenced by the colloquial style, compare *undies* with *panties* and *sunnies* “sunglasses”.

4 *The Age*, Melbourne, October 17, 2002; Corinne Grant on ABC TV’s ‘The Glasshouse’ November 1, 2002; Millwood-Hargrave (2000: 26).

5 Pannick (1985: 145) (English law), and Vagrancy Act, State of Victoria (Australia), §7, 1(c)).

6 See Allan (2001), Fillmore (1982), Schank (1982, 1984, 1986), Schank and Abelson (1977). I find these more satisfactory than the notion of ‘unarticulated constituents’ (Recanati 2002).

7 From the days when booths in public lavatories cost a penny to access.

8 To see how it happened check http://news.bbc.co.uk/sport1/hi/other_sports/us_sport/photo_galleries/3450553.stm. Jackson’s breast is left bare except for a starburst-shaped nipple shield held in place over her aureole by nipple piercing. It is hard to believe that it was indeed a malfunction, i.e. accidental. CBS sustained a record fine of US$550,000 imposed by the Federal Communications Commission for broadcasting this event.

9 ‘Hear how IBM and others are taking up the competitive challenge and improving their structures and processes to enable greater business agility.’ (Monash University MBA Alumni event 19 August 2004).

10 *Letter* originates in a now virtually obsolete verb *let* as in *without let or hinderance* and the tennis expression *let ball* (where the ball catches the net) from the Old English *lettan* meaning “to hinder”. Thus *letter* meant “someone or something which hindered”. This verb *let* came to be homonymous with the more frequent modern English verb *let*, originally *laetan* “to permit, allow” after the latter was subject to regular sound change. Obviously a language cannot easily tolerate homonymous antonyms (auto-antonyms), and the verb with the sense of “hinder” largely disappeared.

11 The French euphemism *j’ai mon anglais* “I’m menstruating” is probably inspired by the redcoats of the English soldiers of yesteryear.

12 Of Diana Crisp September 4, 1660.

13 After an athletic performance with Betty Lane under a tavern chair, January 16, 1664.

14 Of his wife, February 7, 1669. Brought up a Puritan under the Commonwealth, sex was sinful. The view was (and it didn’t change for three hundred years) that virtuous women (and even mistresses) should not take pleasure in sex.

15 April 1, 1667.

16 February 1, 1667. Of Mrs Bagwell, wife of a shipyard carpenter who encouraged his wife’s liaison in order to get business from Pepys.

17 Of Betty Lane in a coach, December 2, 1666.

18 Of Jane Welsh, December 9, 1664 and January 26, 1665.

19 February 9, 1668.

20 Of Jane Turner, September 16, 1668. It is clear from the Diary that maids were commonly abused in this way.

21 Of Deborah Willet, Elizabeth’s maid, March 31, 1668.

22 August 6, 1668.

23 October 25, 1668.

24 August 2, 1660.

25 October 24, 1663.

26 January 1, 1660.

27 December 9, 1663.

28 *Bosom* used to apply to male and female, but seems to have narrowed to (post-pubescent) women alone. This path parallels that of *buxom* which has come to be used of (jolly) women with ample bosoms.

29 Most of the examples of war metaphors mentioned here can be included in the conceptualization sex is war. See Crespo-Fernández (2015).

30 Here are the last five stanzas of Rochester’s *Signior Dildo*:

- This signior is sound, safe, ready, and dumb
  As ever was candle, carrot, or thumb;
  Then away with these nasty devises, and show
  How you rate the just merits of Signior Dildo.
  Count Cazzo [Italian for “prick”], who carries his nose very high,
  In passion he swore his rival should die;
  Then shut up himself to let the world know
  Flesh and blood could not bear it from Signior Dildo.
  A rabble of pricks who were welcome before,
  Now finding the Porter denied ’em the door,
  Maliciously waited his coming below
  And inhumanly fell on Signior Dildo.
  Nigh wearied out, the poor stranger did fly,
  And along Pall Mall they followed full cry;
  The women, concerned, from every window
  Cried, “Oh! For heavens’ sake, save Signior Dildo!”
  The good Lady Sands burst into laughter
  To see how the ballocks came wobbling after,
  And had not their weight retarded the foe,
  Indeed ’t had gone hard with Signior Dildo. (Rochester 1968).

31 Or perhaps *twat* or *twot* meaning “idiot” is sourced in the almost synonymous *twit*. Kate Burridge (pc) says that excrescent*—t* is quite common in English; it is found on *amidst, whilst, betwixt* and *oncest* (many spellings, see the *OED*) once meant “once”. Excrescent *–t* following *n* as in *vermin / varmin* ⇒ *varmint* results from plosive release as the articulators open following the nasal. It is similar to the excrescent *b* in *thimble* ⇐ *þymel* and in *humble* cf. *humility*; and the excrescent final /k/ in some substandard pronunciations of *something* as /^|^sʌmθɪŋk/.

32 On Shakespeare's bawdy see Partridge (1955). For Alice’s adventures, see Carroll (1965).

33 The Slits was an all-female hard rock group (see http://en.wikipedia.org/wiki/The_Slits).

34 The original root means ‘beget’.

35 In Robert Greene’s 1591 book *A Notable Discovery of Coosnage*, cited in Baugh and Cable (1978: 208).

36 Cf. Aman and Sardo (1982: 25). According to Partridge (1970), ‘crumpet’ as in *a bit of crumpet*, i.e. what Americans would denote by *a piece of ass*, is a euphemism which derives from *crumpet* with the sense “buttered bun”.

37 See also Partridge (Partridge 1970: 1351) (probably based on Baker).

38 ‘After the Norman Conquest [the title *earl* was a]pplied to all feudal nobles and princes bearing the Romanic title of Count’ (*OED*).

39 It is possible that *bucket* denotes “beam, yoke” to which an animal was trussed by its hind-legs while its throat was cut. This could be one source for the idiom, but the folk belief has a bucket kicked away as a person hangs.

40 Samuel Collins Epphata to F.T. or the Defence of the Bishop of Elie Concerning his Answer to Cardinall Bellarmine’s Apologue II.x.441, cited in the OED.

41 Some forms dubbed colloquial, e.g. *crick*, are standard for certain English dialects; other ‘colloquial’ forms, e.g. *cuss*, have entered the standard language with a meaning that overlaps the ‘standard’ counterpart but are no longer synonymous with it.

42 On the historical data in the account of *pussy*, check *Oxford English Dictionary*, Allan and Burridge (1991), Grose et al. (1811), Partridge (1970) and Shuy (1987).

43 Script by Graham Chapman, John Cleese, Terry Gilliam, Eric Idle, Terry Jones and Michael Palin; directed by Terry Jones. 1979 Python (Monty) Pictures Ltd.

44 Germanic *tit* is cognate with Romance-based *teat*. It is curious that the latter is apparently never used to denote a breast.
